# Morphological-metabolic analysis in *Streptomyces rimosus* microparticle-enhanced cultivations (MPEC)

**DOI:** 10.1007/s00449-024-03015-2

**Published:** 2024-04-25

**Authors:** Anna Ścigaczewska, Tomasz Boruta, Marcin Bizukojć

**Affiliations:** Faculty of Process and Environmental Engineering, Department of Bioprocess Engineering, Lodz University of Technology, ul. Wolczanska 213, 93-005 Lodz, Poland

**Keywords:** Microparticle-enhanced cultivation, Morphology, MPEC, Secondary metabolites, *Streptomyces*

## Abstract

*Streptomyces* produce a broad spectrum of biologically active molecules such as oxytetracycline and rimocidin, which are widely used in human and animal treatments. microparticle-enhanced cultivation (MPEC) is one of the tools used for *Streptomyces* bioprocesses intensification by the control of mycelial morphology. In the present work, morphological changes of *Streptomyces rimosus* caused by the addition of 10 µm talc microparticles in MPEC were correlated with the biosynthetic activity of the microorganism. Comparing the runs with and without microparticles, major morphological changes were observed in MPEC, including the deformation of pellets, variation of their size, appearance of hyphae and clumps as well as the aggregation of mycelial objects. The presence of talc microparticles also influenced the levels of the studied secondary metabolites produced by *S. rimosus*. Comparing control and MPEC runs, the addition of talc microparticles increased the amounts of oxytetracycline (9-fold), 2-acetyl-2-decarboxamido-oxytetracycline (7-fold), milbemycin A_3_+4[O] (3-fold) and CE 108 (1.5-fold), while rimocidin (27-ethyl) and milbemycin β_11_+4[O] production was reduced. In summary, the addition of talc microparticles to *S. rimosus* cultivations led to the development of smaller morphological forms like hyphae and clumps as well as to the changes in the amounts of secondary metabolites.

**Graphical abstract:**

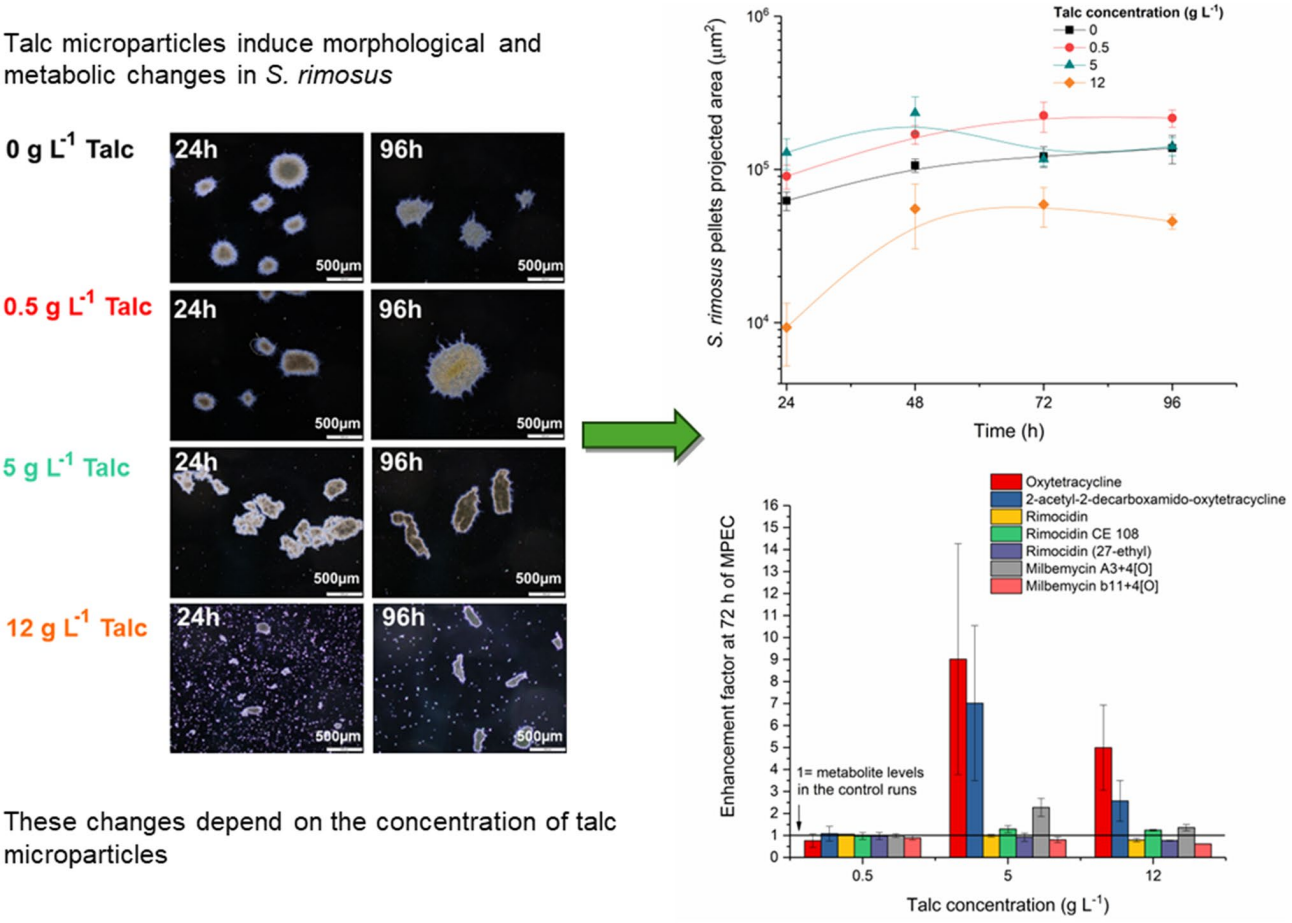

**Supplementary Information:**

The online version contains supplementary material available at 10.1007/s00449-024-03015-2.

## Introduction

The rising problem of microbial resistance to antibiotics and the contemporary high rate of disease spreading trigger the demand for new medicines of natural origin. Many of these compounds can be commercially produced by *actinomycetes*. Their secondary metabolism is an inexhaustible source of bioactive molecules: antibacterial, antiviral, antifungal, antiparasitic and antitumor substances. *Actinomycetes* representing the *Streptomyces* genus are highly relevant in the context of drug manufacturing due to their secondary metabolism encompassing a wide spectrum of biologically active molecules [1]. It is well known that filamentous morphology is one of the key factors influencing the growth and secondary metabolite production in *Streptomyces* cultures [2–5]. Therefore, the importance of seeking new methods to control *actinomycete* morphology in submerged cultivation processes is unquestionable.

Microparticle-enhanced cultivation (MPEC) is a morphological engineering technique employed for the intensification of cultivation processes involving filamentous microorganisms. One of the first studies on the production of *Streptomyces* metabolites in MPEC was conducted by Ren et al. [6]. The addition of 6 µm talc microparticles into *Streptomyces* sp. M-Z18 culture allowed for obtaining 50% higher levels of ε-poly-l-lysine (2.51 ± 0.08 g L^−1^) in comparison to the control experiment without microparticle addition [6]. The most recent studies on morphological engineering techniques with regard to actinomycetes include microparticle-enhanced cultivations (MPEC) of strains from various families [7]. The biosynthesis of seven commercially relevant natural products was boosted by the addition of 10 μm talc microparticles. So far, the highest reported titre of the antibiotic bottromycin A2 and its methylated derivative Met-bottromycin A2 was obtained (109 mg L^−1^) in the MPEC of the recombinant *Streptomyces lividans*. The important observation of the study was that the morphology of *S. lividans* pseudomycelium was physically affected by talc microparticles. Kuhl et al. [7] compared the cultivations without and with the addition of microparticles and concluded that in the presence of talc microparticles *S. lividans* pellets were by 40% smaller, the expression of bottromycin gene cluster was altered, and the morphogenesis was accelerated by the upregulation of developmental regulator genes. The addition of 10 μm talc microparticles was also used in *Streptomyces albus* cultivation to enhance pamamycin production which is an antituberculosis polyketide [8]. The product titre was tripled up to 50 mg L^−1^. The microparticles used in this study affected such morphology-associated regulators as RelA, SsgA, EshA and upregulated to 1024-fold the pamamycin cluster. Summing up, the expression of 56% of all *S. albus* genes (3341 genes) was affected by talc microparticles. The great interest in the cultures of filamentous microorganisms enhanced by microparticles led to the investigation of the effects of the addition of various, chemically distinct microparticles. Walisko et al. [9] tested talc microparticles, surface modified talc microparticles and glass beads in the production of antitumor antibiotics rebeccamycin by *Lechevalieria aerocolonigenes*. The highest titre of the metabolic product in the particle-enhanced shake flask cultivations reached 120 mg L^−1^. Compared to the particle-free *L. aerocolonigenes* control runs, the addition of talc microparticles increased rebeccamycin production threefold and the addition of surface modified talc microparticles resulted in the ninefold enhancement. The addition of different particles to the actinomycete cultures was also tested by Holtmann et al. [10]. They used particles of broken porous SiO_2_ of diameter 120–200 μm (microparticles) and glass beads of diameter 0.25–0.5 mm (macroparticles). The obtained results showed the 85% increase of the level of antibiotic actinorhodin produced by *Streptomyces coelicolor* and the acceleration of streptavidin formation in *Streptomyces avidinii* cultures [10].

The latest review articles discussing morphological engineering of filamentous microorganisms reflected the growing interest in the bioprocess applications of *actinomycetes*. However, the subject of morphology tailoring is still underrepresented and should be investigated by using the novel tools of metabolic engineering and synthetic biology [11, 12]. Surprisingly, only a few studies on morphological engineering with *actinomycete* have been reported so far in the review articles [13, 14]. *S.* *rimosus* was only once mentioned by Kuhl et al. [7] without any specific data. Therefore, the microorganism which was chosen to be the subject for this study was not extensively studied earlier. Nevertheless, its large biosynthetic potential was previously reported [15, 16]. Oxytetracycline, rimocidin and milbemycin produced by *S. rimosus* are widely used in human and animal treatments, both in the bacterial and fungal diseases [17, 18]. The broad spectrum of *S. rimosus* secondary metabolites, including rimocidin CE108, rimocidin (27-ethyl), milbemycin A_3_+4[O], milbemycin β_11_+4[O] was investigated by Boruta et al. [16].

The present work focussed on the morphological changes of *S. rimosus* caused by the addition of talc microparticles in MPEC. These changes were correlated with the biosynthetic outcomes of submerged cultivations, i.e. with the formation of secondary metabolites previously reported in *S. rimosus*: oxytetracycline, 2-acetyl-2-decarboxamido-oxytetracycline, rimocidin, rimocidin CE108, rimocidin (27-ethyl), milbemycin A_3_+4[O] and milbemycin β_11_+4[O].

## Materials and methods

### Strain

*Streptomyces rimosus* ATCC 10970 was studied throughout these experiments. The slants prepared for this microorganism contained Difco™ ISP2 Medium 2 in accordance with the International *Streptomyces* Project guidelines. *S. rimosus* spores were transferred onto the slants and incubated at 26 °C for 5 days to develop its pseudomycelium and induce its sporulation.

### Cultivation method and medium composition

*S. rimosus *shake flasks cultivations with various amounts of talc microparticles added to the basal medium and control experiment without microparticles were made simultaneously. Concentrations of talc microparticles were equal to 0 g L^−1^ (control), 0.5 g L^−1^, 5 g L^−1^, 10 g L^−1^, 12 g L^−1^, making control and four variants of experiments with talc microparticles. The technical details of microparticles supplied by its manufacturer (Termo Scientific, USA) were as follows: white to pale grey talc powder for research and development, 3MgO·4SiO_2_·H_2_O, molecular weight 379.28, particle size − 350 mesh (mean diameter 10 µm), density 2.7 g cm^−3^ at 20 °C, CAS number 14807-96-6.

The basal medium was identical in each variant of the experiment and included yeast extract (5 g L^−1^), glucose (22 g L^−1^), KH_2_PO_4_ (1.50 g L^−1^), MgSO_4_·7H_2_O (0.52 g L^−1^), NaCl (0.4 g L^−1^), ZnSO_4_·7H_2_O (1 mg L^−1^), Fe(NO_3_)_3_·9H_2_O (2 mg L^−1^), biotin (0.04 mg L^−1^), H_3_BO_3_·7H_2_O (0.065 mg L^−1^), MnSO_4_·7H_2_O (0.07 mg L^−1^), CuSO_4_·5H_2_O (0.25 mg L^−1^), Na_2_MoO_4_·2H_2_O (0.05 mg L^−1^). The pH was set on 7 with the use of NaOH solution.

To assure the replicability of the experiments and appropriate growth conditions in each shake flask, namely spore and microparticle concentrations, the following steps were undertaken. First the basal medium was composed. Five flat-bottom flasks each of total volume 500 mL and one Erlenmeyer flask of total volume 300 mL were made out for each talc concentration run and for the control. A 120 mL of the basal medium was poured into all flat-bottom flasks (cultures preparation) and a 150 mL into the one Erlenmeyer flask (inoculum preparation). The appropriate amounts of talc calculated so that to give the assumed final microparticle concentration were weighed out into 50 mL beakers and secured with aluminium foil. In the next step all flasks with the basal medium and beakers with dry talc microparticles were autoclaved at 121 °C for 30 min. Inoculum was prepared by washing *S. rimosus* slant into the aforementioned sterilised Erlenmeyer flask. After mixing it with the selected amount of talc microparticles, a 30 mL of the inoculum was poured into each of the five flat-bottom flasks provided for one run of the experiment. For the control run the microparticle addition step was skipped. The final volume of the medium in each shake flask during the cultivation was equal to 150 mL (120 mL of the medium + 30 mL of the inoculum with or without talc addition).

Spore concentration in the inoculum was set on 10^9^ per litre based on Thoma cell counting chamber measurements. Inoculated flasks with and without talc were cultivated for 96 h at 28 °C in an orbital shaker Certomat® BS-1 (B. Braun Biotech International, Berlin, Germany) at 110 rpm. During the experiments the samples were collected every 24 h including 0 h. Each experiment consisting of the control run and four runs with various talc concentrations was replicated four times.

### Morphological analysis and semiautomatic image processing

Morphological analysis was carried out using the light phase contrast microscope (OLYMPUS BX53, Olympus Corporation, Japan), high-resolution RGB digital camera (OLYMPUS DP27) and image analysis software (OLYMPUS cellSens Dimension Desktop 1.16, Olympus Corporation, Japan). Microscopic images for the digital image analysis were snapped during microscopic observations of the morphological objects found in the samples collected every 24 h of the experiments. The median filter and Sobel filter were applied before segmentation in the semiautomatic image processing procedure. Morphological parameters including projected area (*A*), elongation (*E*) and roughness (*R*) were calculated during the analysis of the snapped image. They were then used for morphology number (Mo) calculations. Projected area (*A*) describes the size of the studied objects, elongation (*E*) and roughness (*R*) parameters determine their shape. The terms of the morphological parameters are defined in OLYMPUS cellSens Dimension Desktop 1.16 as follows:

- Projected area (*A*)—the number of pixels contained in an object multiplied by the squared calibration unit;
- Elongation (*E*)—the squared quotient of longitudinal and transversal deviation of all pixels belonging to the object along the regression line;
- Roughness (*R*)—the area relative to the area of the object’s convex hull.

Morphology number (Mo) was calculated in accordance with Wucherpfennig et al. [19].

$${\text{Mo}} = \frac{2 \cdot \sqrt A \cdot S}{{\sqrt \pi \cdot D \cdot E}}$$

*A*—projected area, *S*—solidity, identical regarding the definition with the roughness *R*, *D*—maximum diameter of the object, *E*—elongation.

Morphology number combines the size and the shape parameters giving information about objects morphology. Its values are within the range from 0 to 1. For circular, regular objects like pellets or spores morphology number approaches 1. For the elongated hyphae or irregular clump forms morphology number decreases.

The detailed description of the morphological analysis, semiautomatic image processing and the morphological parameters can be found in the previously published papers [20, 21].

### Analysis of secondary metabolites and glucose concentrations

The secondary metabolites present in the culture broth were analysed with the use of the ultra-high performance liquid chromatography (UPLC® Acquity, Waters, USA) coupled with high resolution mass spectrometry (ACQUITY-SYNAPT G2, Waters, USA) The analysis of secondary metabolites was conducted in the positive and negative electrospray ionisation modes (ESI^+^ and ESI^−^). Detailed procedure can be found in [16]. The secondary metabolites identification was carried out on the basis of the analytical standards (oxytetracycline) and database of metabolites: the Natural Products Atlas [https://www.npatlas.org/]. The quantitative and semi-quantitative analyses of the secondary metabolites were conducted with the use of TargetLynx software (Waters, USA).

Glucose concentration was determined on a UPLC BEH Amide column (Waters, USA) of dimensions (2.1 mm × 150 mm × 1.7 μm) at temperature 35 °C and eluent flow rate 0.29 mL min^−1^. Eluent contained 75% acetonitrile solution with 0.2% of triethylamine in deionised water. An evaporated light scatter detector (ELS) was used to quantify the amount of glucose. Prior to all chromatographic analyses, the samples were filtered through 0.2 µm syringe filters.

### Glucose uptake rate calculations

PTC Mathcad 15 software was used to calculate glucose uptake rates. The experimental data (glucose concentration changes in time) were approximated by cubic b-spline function. In the next step, the approximation function was differentiated to find the changes of volumetric glucose uptake rate in time.

### Statistical analysis

Mean values of morphological parameters were calculated using 30 or more actinomycete objects. Statistical analysis of secondary metabolite enhancement factors and substrate (glucose) concentrations were conducted on the basis of four replications of the experiments. It provided statistically significant data for calculating standard deviation and confidence bands with the significance level *α* = 0.05. *P*-values were generated by statistical *t*-test and if they were lower than 0.05, it confirmed the statistical significance of the differences for the values being compared during the elaboration of results.

## Results

**Fig. 1:**
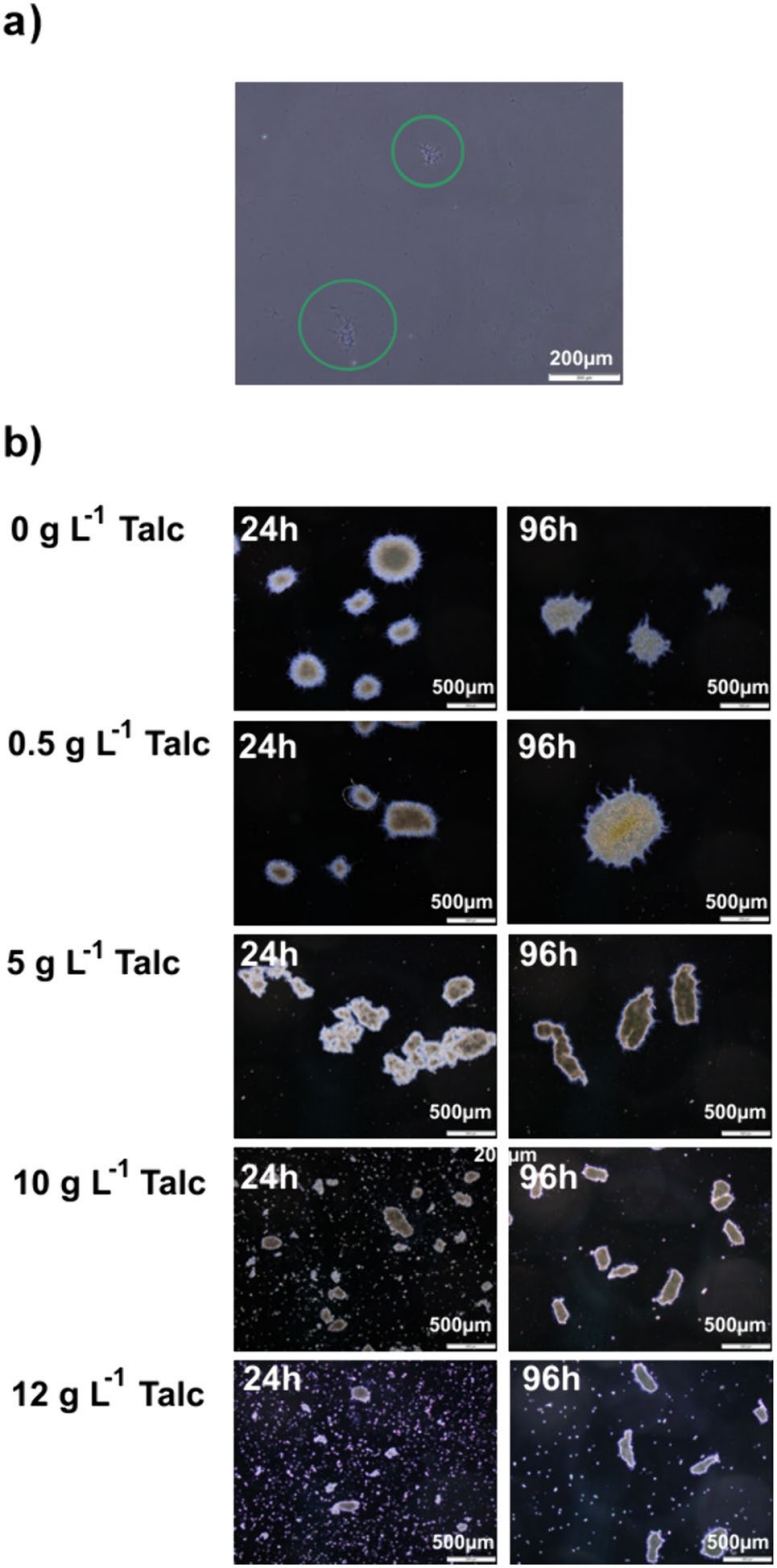
Images of *S. rimosus* morphological forms: **a** Exemplary hyphae and clumps in the run with 0.5 g L^−1^ of talc at 96 h, scale 200 µm; **b** pellets in 24 h and 96 h of the control run and of the runs with 0.5, 5, 10 and 12 g L^−1^ of talc microparticles, scale 500 µm

*S. rimosus s*hake flasks experiments were conducted to determine the effect of the addition of talc microparticles on this microorganism cultivated under submerged conditions. *S. rimosus* morphological forms observed during the experiments were classified into two morphological groups of objects. The first group comprised elongated hyphae and loosely compacted clump forms (Fig. 1a). In the second group, there were pellets with well-evolved structure and higher values of projected area A in comparison to the objects from the first group (Fig. 1b). The microscopic images of the morphological objects presented in Fig. 1b also show the changes of pellets structure caused by the increasing concentrations of talc microparticles. In 24 h of the control run (without microparticles), pellets were round and regular, however, at the end of the experiment they underwent slight deformation. In the next run, with the smallest amount of talc microparticles (0.5 g L^−1^), microparticles were embedded into the pellets, strengthening their structure (Fig. 1b). In the run with 5 g L^−1^ of talc microparticles the shape of pellets changed markedly. They were deformed to the elongated, irregular objects which also agglomerated with each other. The addition of two highest amounts of talc microparticles (10 and 12 g L^−1^) resulted in the similar elongation of pellets, nevertheless the presence of talc suspended in the culture medium prevented the agglomeration of the objects and further increase of their size (Fig. 1b). All these observed morphological changes were quantitatively described using the morphological parameters.

**Fig. 2:**
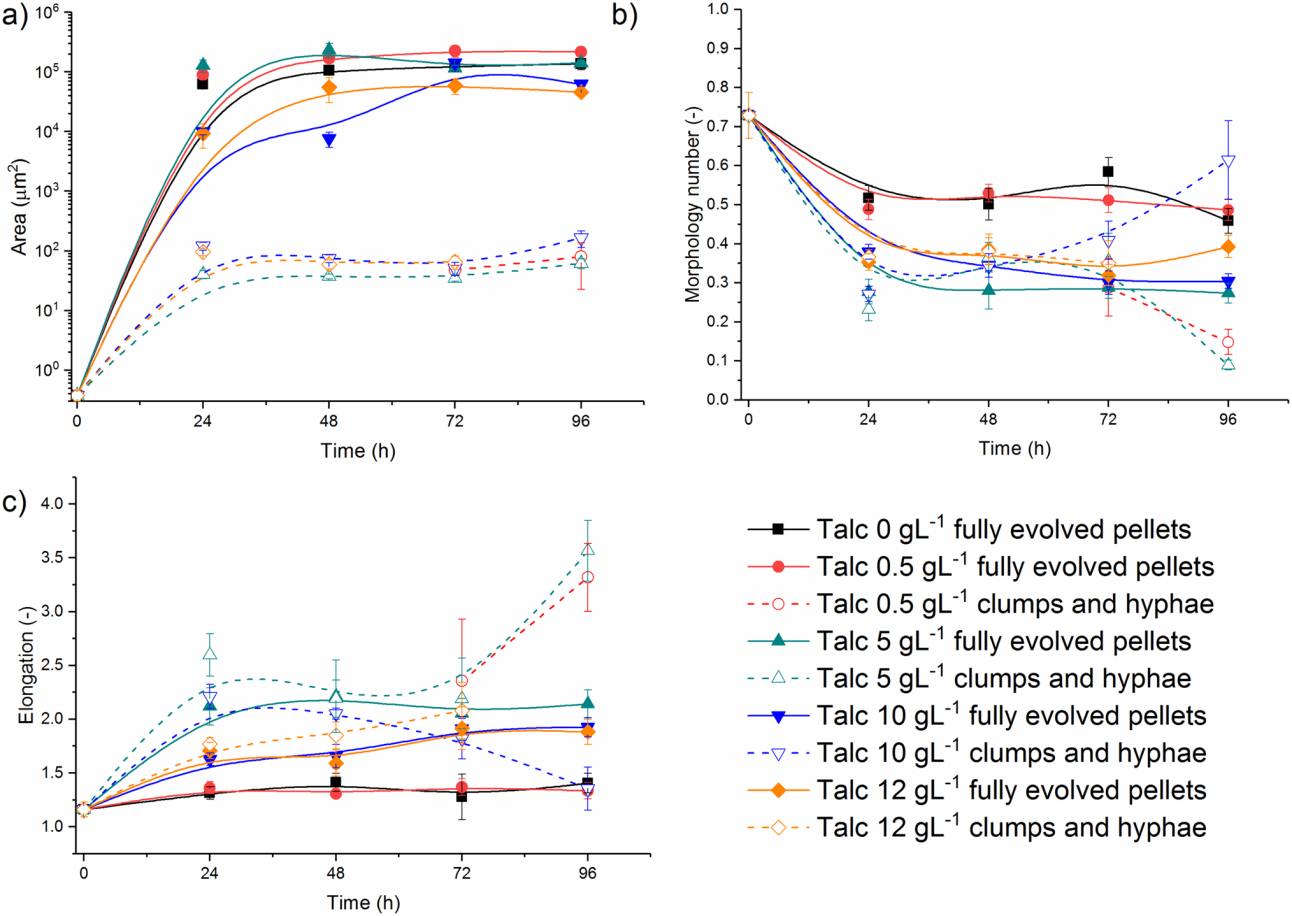
Evolution of morphological forms of *S. rimosus* based on projected area *A* (**a**), morphology number Mo (**b**) and elongation *E* (**c**) changes in time

The course of the morphological changes occurring during the experiments is shown in Fig. 2. After the first 24 h of growth, *S. rimosus* pellets of projected area *A* in the range from 10^4^ to 10^6^ µm^2^ were formed, regardless of talc microparticles concentration in the medium (solid markers in Fig. 2a). The smaller morphological forms, clumps and hyphae with projected area not exceeding 164 µm^2^, were found in all runs with the addition of talc (hollow markers in Fig. 2a). The exception was the control run without microparticles (Fig. 2), in which clump forms and loose hyphae were not observed.

The values of morphology number Mo (Fig. 2b) and elongation *E* (Fig. 2c) for the morphological group of *S. rimosus* pellets and for the morphological group of clumps and hyphae were not so distant from each other, as opposed to their projected area values (Fig. 2a). The most circular objects with elongation equal to 1.24 and regular round shape indicated by morphology number 0.74 were *S. rimosus* spores found in 0 h of the experiment. During next 24 h of cultivation the morphology number decreased to 0.52 (the highest value obtained for pellets, in the run without talc microparticles) and 0.23 (the lowest value obtained for clumps and hyphae, in the run with 5 g L^−1^ of talc). Noteworthy observations were made for the objects from the run with 12 g L^−1^ of talc microparticles. Although the projected area for the morphological group of pellets and for the morphological group of clumps and hyphae in 24 h differed by two orders of magnitude (Fig. 2a), their morphology numbers were almost identical, equal to 0.35 and 0.37, respectively. What is more, the *P*-value lower than 0.0001 indicated statistically significant differences between these two morphology number values (Fig. 2b). In the end of this run, the group of clumps and hyphae vanished due to hyphal agglomeration and only one morphological group, namely group of pellets, remained. What is more, in the run with the second highest talc concentration of 10 g L^−1^, the group of clumps and hyphae did not agglomerate into pellets as it was recorded in the run with 12 g L^−1^ of talc. Nevertheless, their morphology number increased to 0.61 in 96 h, thereby reaching the highest value (excluding spores at 0 h). Therefore, the morphology of the group of clumps and hyphae was influenced by 10 g L^−1^ of talc microparticles in such a manner that it maintained their small sizes of 10^2^ µm^2^. The values of elongation (Fig. 2c) indicated that the most elongated objects were formed in the end of the run with 5 g L^−1^ of talc and 0.5 g L^−1^ of talc. Elongation *E* values for the group of clumps and hyphae at 96 h reached 3.57 and 3.32 respectively. At the same time, elongation *E* for the pellets were equal to 2.14 in the run with 5 g L^−1^ of talc and 1.34 in the run with 0.5 g L^−1^ of talc. For the hyphae and clumps in the run with 10 g L^−1^ of talc the course of elongation changes was modified by the addition of microparticles. After its preliminary increase lasting up to 24 h, this parameter began to decrease to the final value of 1.35 in 96 h.

**Fig. 3:**
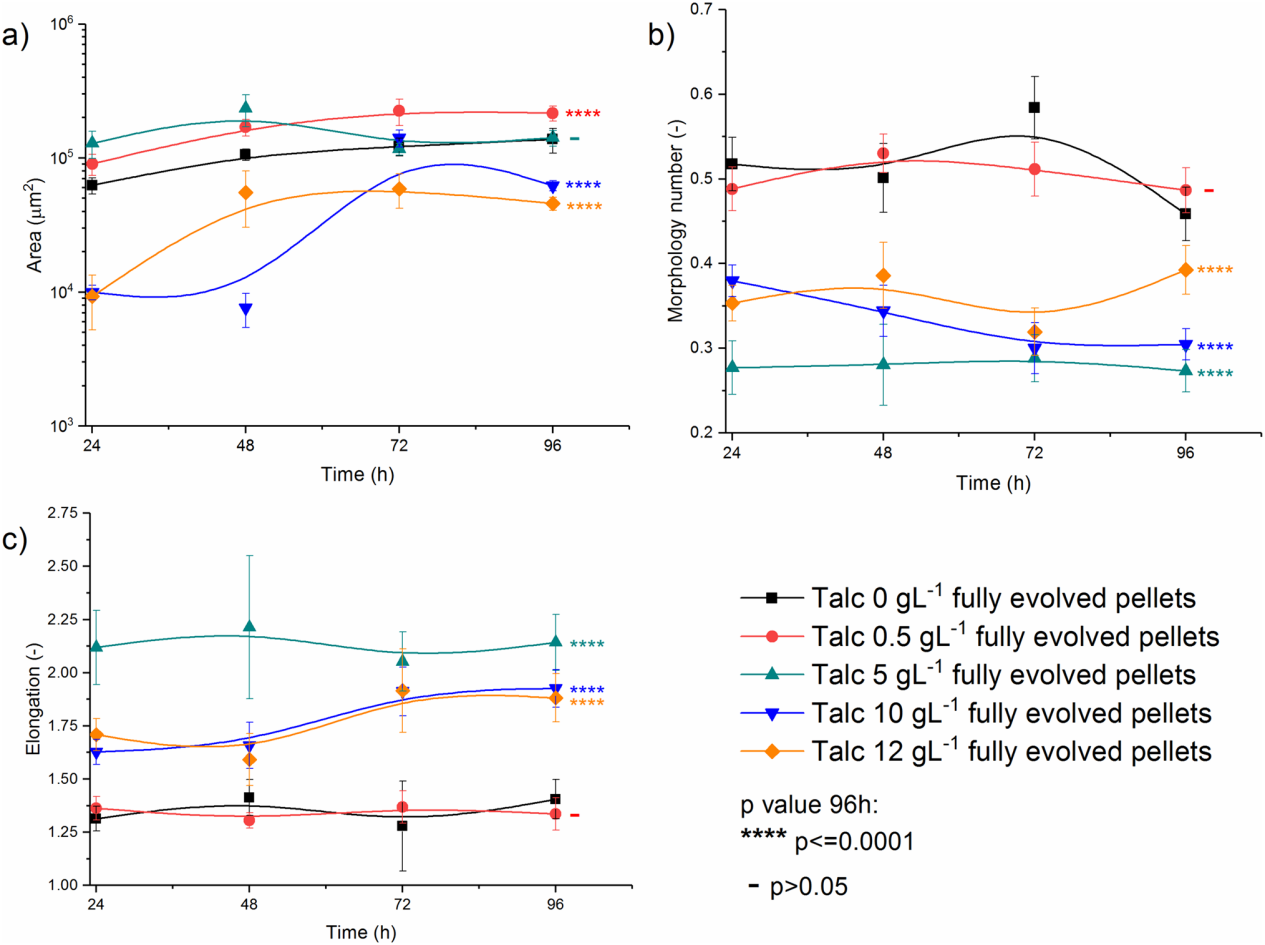
Evolution of *S. rimosus* pellets based on projected area *A* (**a**), morphology number Mo (**b**) and elongation *E* (**c**) changes in time

The influence of talc microparticles addition on the morphology group of *S. rimosus* pellets is shown in detail in Fig. 3. Morphological parameters (*A*, Mo, *E*) for the pellets evolved after 24 h indicated on the morphological differences in the runs with and without microparticles. *P*-values at 96 h of the control run without talc and in the runs with the chosen amount of talc were calculated for the sake of clarity (Fig. 3).

The values of projected area for the control run in 72 h and 96 h, when the production of secondary metabolites was the highest, were equal to 1.2 × 10^5^ µm^2^ and 1.4 × 10^5^ µm^2^, respectively. Identical values (and *P* > 0.05 in 96 h) were obtained for the run with 5 g L^−1^ of talc, despite the fact that earlier in this run (24 h and 48 h) the highest projected area out of all MPEC runs was observed (Fig. 3a). For the other runs with the addition of talc the differences between the values of projected area of pellets and its respective values from the control run were significant (*P* < 0.0001 in 96 h, Fig. 3a). In the run with the lowest microparticle concentration, 0.5 g L^−1^ of talc, the pellets grew larger in comparison to the control and in the end their projected area was equal to 2.2 × 10^5^ µm^2^. In the remaining two runs, with the highest talc concentrations 10 g L^−1^ and 12 g L^−1^, the pellets were smaller than the corresponding objects from the control run. The final projected area reached 6.2 × 10^4^ µm^2^ (10 g L^−1^ of talc) and 4.6 × 10^4^ µm^2^ (12 g L^−1^ of talc).

Although the size of pellets in the run with 5 g L^−1^ of talc expressed as projected area was the same as in the control run (Fig. 3a), their shapes were different in comparison to the run without the talc addition (Fig. 3b, c). The morphology number Mo of pellets in 96 h of the run with 5 g L^−1^ of talc was equal to 0.27, while the corresponding value in the control reached 0.46. The highest morphology number Mo at 96 h was equal to 0.49, and hence the most regular and circular shapes were obtained for the pellets from the run with 0.5 g L^−1^ of talc. However, the difference between this value of morphology number and the respective one from the control run was not statistically significant (Fig. 3b). Summing up, in the case of *S. rimosus* pellets the addition of the lowest talc amount equal to 0.5 g L^−1^ led to the increase of the final morphology number as well as projected area (Fig. 3a, b). Moreover, higher talc concentrations caused the decrease of morphology number, which in the runs with 10 g L^−1^ and 12 g L^−1^ of talc went hand in hand with the smaller projected area values (Fig. 3a, b).

The changes of pellets elongation (Fig. 3c) were consistent with their morphology number Mo. In the case of the run with the lowest morphology number (5 g L^−1^ of talc), elongation reached the highest value of 2.14 indicating the most elongated morphological objects. The lowest elongation was found in the control run (1.41) and in the run with 0.5 g L^−1^ of talc (1.34) and the difference between them was not significant (Fig. 3c). The pellets obtained in the runs with 10 g L^−1^ and 12 g L^−1^ of talc were more elongated in comparison to the control and their elongations were equal to 1.93 and 1.88, respectively.

**Fig. 4:**
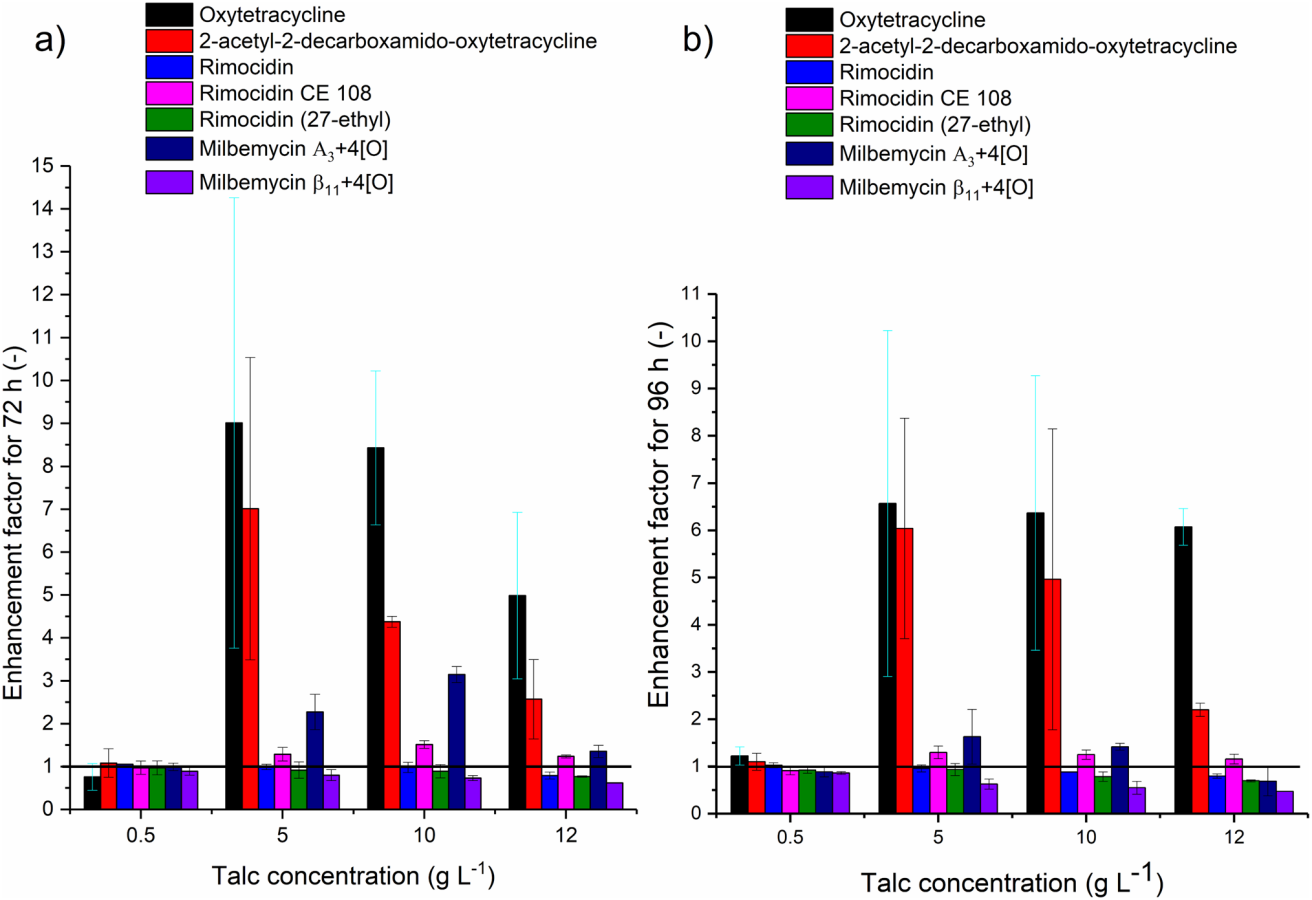
Enhancement factors calculated for oxytetracycline, 2-acetyl-2-decarboxamido-oxytetracycline, rimocidin, rimocidin CE108, rimocidin (27-ethyl), milbemycin A_3_+4[O], milbemycin β_11_+4[O] in the 72 h of the experiments (**a**) and in the 96 h of the experiments (**b**)

The addition of talc microparticles influenced the production of the selected *S. rimosus* secondary metabolites. It was presented as enhancement factors (Fig. 4). The enhancement factors (EF) were calculated as mean amount of metabolite obtained in the run with talc microparticles divided by the corresponding value in the control run:

$${\text{EF = }}\frac{{\text{mean amount of a metabolite for the run with talc microparticles}}}{{\text{mean amount of a metabolite for the control run}}}$$

Oxytetracycline concentrations were analysed quantitatively and the amounts of other metabolites were expressed in terms of their [M + H]^+^ or [M−H]^−^ ion peak areas. The structures of the detected and studied *S. rimosus* secondary metabolites and their experimental monoisotopic masses at ESI^−^ ionisation are presented in the Supplementary materials (Figs. S1–S7). The most remarkable influence of talc microparticles on *S. rimosus* cultures was observed in the case of oxytetracycline and 2-acetyl-2-decarboxamido-oxytetracycline both in 72 h (Fig. 4a) and 96 h (Fig. 4b) of the experiments. In 72 h of the run with 5 g L^−1^ of talc (Fig. 4a) the mean oxytetracycline amount was ninefold higher compared to the control run without microparticles. Moreover, in the run with 5 g L^−1^ of talc the highest oxytetracycline concentration equal to 9.5 mg L^−1^ was obtained. The corresponding value from the control run reached only 0.7 mg L^−1^. Not much lower was the enhancement factor obtained for 2-acetyl-2-decarboxamido-oxytetracycline in 72 h of the run with 5 g L^−1^ of talc (Fig. 4a). The highest 2-acetyl-2-decarboxamido-oxytetracycline amount reached 279 [a.u.] for the run with 5 g L^−1^ talc and it was sevenfold higher that in the particle-free run. The noteworthy enhancement factor exceeding the value of 3 was noted for milbemycin A_3_ + 4[O] in 72 h of the run with 10 g L^−1^ of talc (Fig. 4a). The compound rimocidin CE 108 was also enhanced by the addition of talc microparticles (Fig. 4a, b). Its highest enhancement factor was equal to 1.5 (72 h, 10 g L^−1^ of talc). The levels of rimocidin (27-ethyl) and milbemycin β_11_ + 4[O] were decreased by talc addition to *S. rimosus* cultivations and rimocidin formation was not influenced by the presence of microparticles (Fig. 4a, b).

**Fig. 5:**
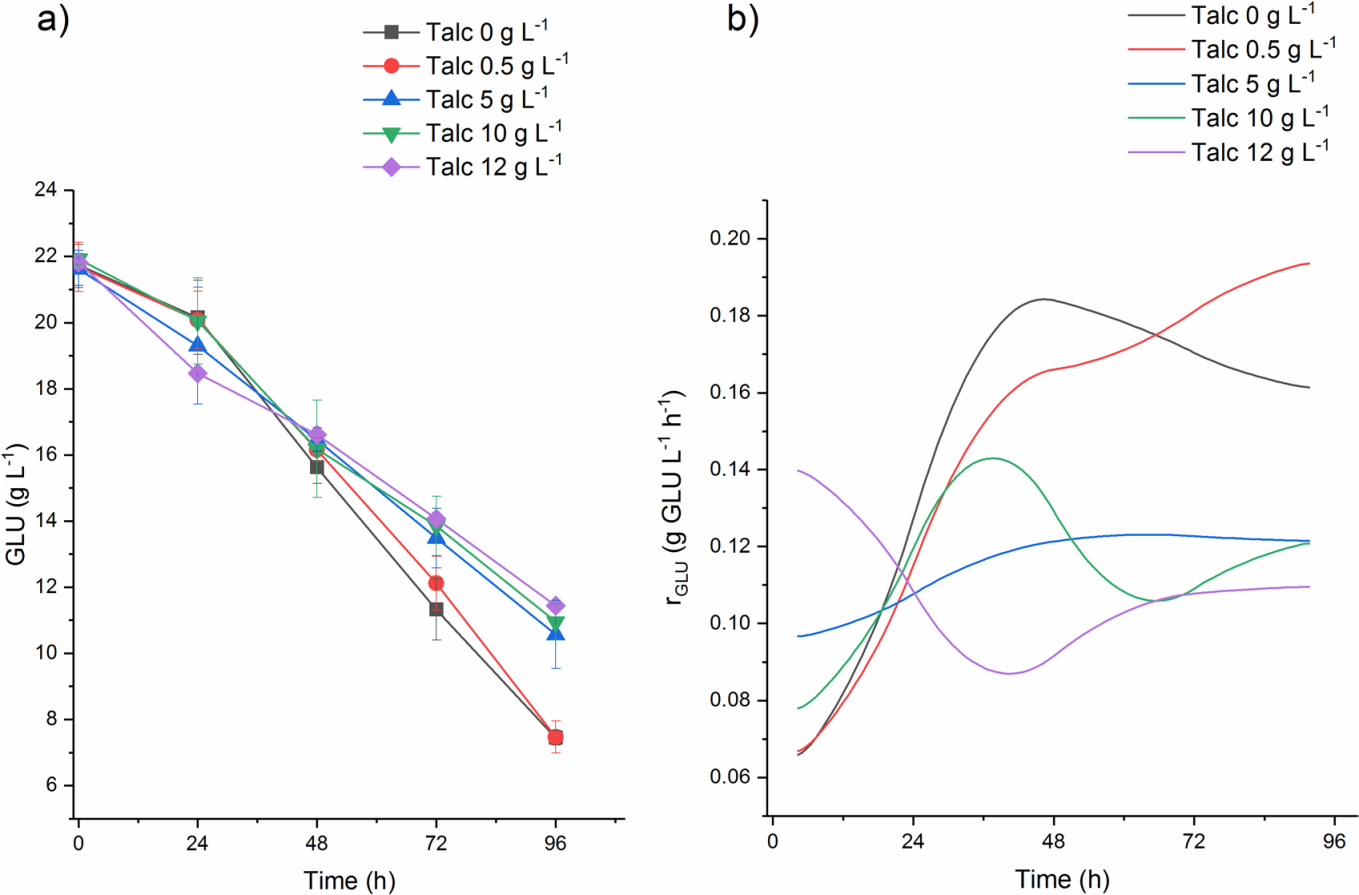
The changes of glucose concentration (**a**) and glucose uptake rate (**b**) during *S. rimosus* cultivations with and without talc microparticles addition

The concentration of glucose (Fig. 5a) being the main carbon source in the culture medium was analysed during the experiments. The obtained data allowed for the calculation of glucose uptake rate (Fig. 5b). Comparing the influence of all examined talc concentrations on *S. rimosus* culture, the final mean values (96 h of the experiments) of glucose concentration obtained from four replications were significantly lower for two runs: control without talc and the run with 0.5 g L^−1^ of talc. Their exact values were 7.45 and 7.47 g L^−1^, respectively. The amount of the unutilized glucose increased along the concentration of talc microparticles in the culture medium (Fig. 5a). In 96 h of the run with 5 g L^−1^ of talc glucose concentration was equal to 10.56 g L^−1^, in the run with 10 g L^−1^ of talc it reached 10.95 g L^−1^. In the run with the highest tested talc concentration (12 g L^−1^) glucose amount was at the level of 11.44 g L^−1^. Changes of glucose uptake rate demonstrated the same pattern as glucose concentration profiles (Fig. 5b). Despite of the lowest value at the beginning of control run and the run with 0.5 g L^−1^ of talc microparticles (equal to 0.07 g GLU L^−1^ h^−1^), glucose uptake rates in theses runs were increasing rapidly until 48 h and remained on the relatively high level until the end of the experiment (Fig. 5b). Glucose uptake rates at the end of the control and the run with 0.5 g L^−1^ of talc were equal to 1.16 g GLU L^−1^ h^−1^ and 1.19 g GLU L^−1^ h^−1^, respectively. For the sake of comparison, glucose uptake rates in 96 h of the runs with the higher talc concentrations equal to 5, 10 and 12 g L^−1^ were much lower and had the respective values of 0.12, 0.12, and 0.10 g GLU L^−1^ h^−1^.

## Discussion

The influence of microbial morphology on the production of secondary metabolites by filamentous microorganisms (actinomycetes and fungi) has been reported many times in previous studies [inter alia 2, 3, 14, 22]. Therefore, morphological engineering and techniques related to it, to which microparticle-enhanced cultivation (MPEC) belongs, aroused a great interest in the last 20 years [23]. It was proved that the morphological objects that allow for the efficient mass transfer were small pellets [24], “hairy” agglomerates, clump forms and loose hyphae [14]. Therefore, reducing the size of morphological objects by adding microparticles may increase the efficiency of metabolite biosynthesis by filamentous microorganisms [25]. One of the first applications of MPEC for *Streptomyces* which resulted in the enhancement of metabolite production connected with the morphological changes was conducted by Ren et al. [6]. This research group introduced 6 µm talc microparticles into *Streptomyces* sp. M-Z18 culture and obtained up to 50% higher levels of ε-poly-l-lysine (2.51 ± 0.08 g L^−1^) in comparison to the control experiment. This enhancement of actinomycete metabolism was attributed to the decrease of pellet diameter caused by the addition of talc microparticles. In our study, the similar dependence was observed and the effect of microparticles on a *Streptomyces* species. Comparing *S. rimosus* pellets from the runs with and without microparticles, it was found that the addition of higher talc concentrations at 10 g L^−1^ and 12 g L^−1^ to the cultivations led to the reduction of projected area of pellets (Fig. 3a) and caused to the decrease of morphology number Mo (Fig. 3b). Projected area *A* in 96 h of the experiments reached 6.2 × 10^4^ µm^2^ (10 g L^−1^ of talc) and 4.6 × 10^4^ µm^2^ (12 g L^−1^ of talc) while in the control run it was 1.4 × 10^5^ µm^2^ (*P* < 0.0001). What is more, the smaller morphological forms, clumps and hyphae, appeared only in the runs with the addition of talc (Fig. 2a). The addition of talc microparticles influenced not only the size of the *S. rimosus* objects, but also caused to a change in their shapes. Elongation of *S. rimosus* pellets increased (Fig. 3c), hence the developed objects were not only smaller but more irregular either (Fig. 1). Shape changes of the morphological objects caused by microparticles addition were also reported in the literature. Filamentous fungi species with various mechanisms of pellets formation were examined [26]. For example in *C. globosum* submerged cultivations Al_2_O_3_ microparticles addition remarkably changed the shapes of pellets from the circular to the star-shaped one [26].

Nevertheless, the addition of microparticles would not always reduce the size of the objects, which was often overlooked by researchers. Kowalska et al. [20] tested various filamentous fungal species in MPEC with Al_2_O_3_ microparticles and proved that their addition may cause agglomeration of morphological forms leading to the development of larger agglomerates. The increase of the size of mycelial objects by Al_2_O_3_ microparticles addition was obtained for a non-agglomerative *M. racemosus* while for the agglomerative *A. terreus* the results were opposite. Therefore, the final outcome of the application of MPEC is related to the specific microorganism morphology development mechanism either agglomerative or non-agglomerative [20]. Apart from it, the physical forces occurring between mycelial objects (spores, hyphae, pellets) and microparticles including electrostatic and van der Waals forces, steric interactions and hydrophobicity of the elements suspended in the culture medium also play the important role in the morphological development of the given species [13]. In this study, the addition of lower amounts of talc microparticles (on the level of 0.5 g L^−1^–5 g L^−1^) increased the projected area of *S. rimosus* pellets as well as their morphology number Mo (Fig. 3a, b). The exact size of the pellets in 96 h of the run with talc 0.5 g L^−1^ was equal to 2.2 × 10^5^ µm^2^ and corresponding value for pellets in the control run reached 1.4 × 10^5^ µm^2^ (*P* < 0.0001). These data indicate that *S. rimosus* pellets that were developing in the run with talc 0.5 g L^−1^ had circular shapes and their sizes were larger in comparison to the control run and to the runs with higher talc concentrations.

The morphological results were confronted with the analysis of the amounts of secondary metabolites produced by the studied actinomycete. The occurrence of smaller morphological forms as hyphae and clumps in the runs with talc microparticles caused the changes in the amount of secondary metabolite formed during the experiments. Oxytetracycline, 2-acetyl-2-decarboxamido-oxytetracycline, milbemycin A_3_+4[O] and rimocidin CE 108 amounts increased, while rimocidin (27-ethyl) and milbemycin β_11_+4[O] production was reduced by the addition of talc microparticles (Fig. 4a, b). The most remarkable influence of talc addition to the *S. rimosus* cultures was observed for oxytetracycline (its amount was up to ninefold higher comparing to the control run without microparticles) and for 2-acetyl-2-decarboxamido-oxytetracycline (sevenfold higher). Comparable results were obtained by Walisko et al. [9]. The addition of surface modified talc microparticles to the *L. aerocolonigenes* cultivation increased rebeccamycin production ninefold. What is more, apart from their morphological and metabolic influence, microparticles are thought to induce genetic modifications in the gene clusters and morphogenesis [7, 8] In the research conducted by Kuhl et al. [8] the expression of 56% of all *S. albus* genes (3341 genes) was affected by the microparticle addition. In *S. lividans* cultivations [7] pellets grew by 40% smaller, the expression of bottromycin cluster gene was modified and morphogenesis was accelerated by the upregulation of the developmental regulator genes.

Summing up, comparing the *S. rimosus* runs without microparticles and MPEC major differences were observed that can be concluded in the following statements. Pellet shape deformation and their variation of size are observed in *S. rimosus* MPEC. Moreover, hyphae and clump forms appear due the action of microparticles. The addition of talc microparticles does not always reduce the size of *S. rimosus* objects. At lower talc concentrations (from 0.5 g L^−1^ to 5 g L^−1^) added to *S. rimosus* culture the agglomeration of morphological forms takes place, leading to the development of larger agglomerates. The decrease of *S. rimosus* pellet size takes place only for higher (10 g L^−1^ and 12 g L^−1^) talc microparticle concentrations. The presence of talc microparticles has the undeniable effect on the levels of several secondary metabolites of *S. rimosus*. Oxytetracycline, 2-acetyl-2-decarboxamido-oxytetracycline, milbemycin A_3_+4[O] and rimocidin CE 108 production is enhanced in MPEC, while rimocidin (27-ethyl) and milbemycin β_11_+4[O] production is reduced.

MPEC technique in the actinomycete cultivations occurred to be an effective tool for morphology tailoring. Based on our current knowledge, microparticles can be easily applied in bioprocesses involving filamentous microorganisms for the improvement of secondary metabolite production. Furthermore, morphological and genetic analysis of filamentous microorganisms in MPEC could provide fundamental knowledge on their growth and development. It is especially important for *Streptomyces* species which is still underrepresented in the studies on tailored morphology.

### Supplementary Information

Below is the link to the electronic supplementary material.

Supplementary file1 (PDF 151 KB)

## Data Availability

The data that support the findings of this study are available from the corresponding author, Anna Ścigaczewska, upon reasonable request.

## Declarations

### Research involving human and animals participants

This article does not contain any studies with human participants or animals performed by any of the authors.

## References

[1] Pommerehne K, Walisko J, Ebersbach A, Krull R (2019). The antitumor antibiotic rebeccamycin-challenges and advanced approaches in production processes. Appl Microbiol Biotechnol.

[2] Chater KF (1984). Morphological and physiological differentiation in *Streptomyces*. Microb Dev.

[3] Dobson LF, O’Cleirigh CC, O’Shea DG (2008). The influence of morphology on geldanamycin production in submerged fermentations of *Streptomyces hygroscopicus *var. *geldanus*. Appl Microbiol Biotechnol.

[4] van Wezel GP, Krabben P, Traag BA, Keijser BJF, Kerste R, Vijgenboom E, Heijnen JJ, Kraal B (2006). Unlocking *Streptomyces* spp. for use as sustainable industrial production platforms by morphological engineering. Appl Environ Microbiol.

[5] Yue C, Xu H, Yu Y, Yu X, Yu M, Zhang C, You Q, Xia S, Ding Z, Fu H, Zeng X, Li F (2021). Improvement of natamycin production by controlling the morphology of *Streptomyces gilvosporeus* Z8 with microparticle talc in seed preculture. JCTB.

[6] Ren XD, Xu YJ, Zeng X, Chen XS, Tang L, Mao ZG (2015). Microparticle-enhanced production of ε-poly-l-lysine in fed-batch fermentation. RSC Adv.

[7] Kuhl M, Rückert C, Gläser L, Beganovic S, Luzhetskyy A, Kalinowski J, Wittmann C (2021). Microparticles enhance the formation of seven major classes of natural products in native and metabolically engineered actinobacteria through accelerated morphological development. Biotechnol Bioeng.

[8] Kuhl M, Gläser L, Rebets Y, Rückert C, Sarkar N, Hartsch T, Kalinowski J, Luzhetskyy A, Wittmann C (2020). Microparticles globally reprogram *Streptomyces albus* toward accelerated morphogenesis, streamlined carbon core metabolism, and enhanced production of the antituberculosis polyketide pamamycin. Biotechnol Bioeng.

[9] Walisko J, Vernen F, Pommerehne K, Richter G, Terfehr J, Kaden D, Dähne L, Holtmann D, Krull R (2017). Particle-based production of antibiotic rebeccamycin with *Lechevalieria aerocolonigenes*. Process Biochem.

[10] Holtmann D, Vernen F, Müller JM, Kaden D, Risse JM, Friehs K, Dähne L, Stratmann A, Schrader J (2017). Effects of particle addition to *Streptomyces* cultivations to optimize the production of actinorhodin and streptavidin. Sustain Chem Pharm.

[11] Karahalil E, Coban HB, Turhan I (2018). A current approach to the control of filamentous fungal growth in media: microparticle enhanced cultivation technique. Crit Rev Biotechnol.

[12] Böl M, Schrinner K, Tesche S, Krull R (2020). Challenges of influencing cellular morphology by morphology engineering techniques and mechanical induced stress on filamentous pellet systems—a critical review. Eng Life Sci.

[13] Laible AR, Dinius A, Schrader M, Krull R, Kwade A, Briesen H, Schmideder S (2021). Effects and interactions of metal oxides in microparticle-enhanced cultivation of filamentous microorganisms. Eng Life Sci.

[14] Lajtai-Szabó P, Hülber-Beyer É, Nemestóthy N, Bélafi-Bakó K (2022). The role of physical support in secondary metabolite production by *Streptomyces species*. Biochem Eng J.

[15] Jonsbu E, McIntyre M, Nielsen J (2002). The influence of carbon sources and morphology on nystatin production by *Streptomyces noursei*. J Biotechnol.

[16] Boruta T, Ścigaczewska A, Bizukojć M (2021). "Microbial wars" in a stirred tank bioreactor: investigating the co-cultures of *Streptomyces rimosus* and *Aspergillus terreus*, filamentous microorganisms equipped with a rich arsenal of secondary metabolites. Front Bioeng Biotechnol.

[17] Pickens LB, Tang Y (2010). Oxytetracycline biosynthesis. JBC.

[18] Boruta T (2021). A bioprocess perspective on the production of secondary metabolites by *Streptomyces* in submerged co-cultures. World J Microbiol Biotechnol.

[19] Wucherpfennig T, Hestler T, Krull R (2011). Morphology engineering-osmolality and its effect on *Aspergillus niger* morphology and productivity. Microb Cell Fact.

[20] Kowalska A, Boruta T, Bizukojć M (2018). Morphological evolution of various fungal species in the presence and absence of aluminum oxide microparticles: comparative and quantitative insights into microparticle-enhanced cultivation (MPEC). Microbiologyopen.

[21] Ścigaczewska A, Boruta T, Bizukojć M (2021). Quantitative morphological analysis of filamentous microorganisms in cocultures and monocultures: *Aspergillus terreus* and *Streptomyces rimosus* warfare in bioreactors. Biomolecules.

[22] Yin P, Wang YH, Zhang SL, Chu J, Zhuang YP, Chen N, Li XF, Wu YB (2008). Effect of mycelial morphology on bioreactor performance and avermectin production of *Streptomyces avermitilis* in submerged cultivations. J Chin Inst Chem Eng.

[23] Kaup BA, Ehrich K, Pescheck M, Schrader J (2008). Microparticle-enhanced cultivation of filamentous microorganisms: increased chloroperoxidase formation by *Caldariomyces fumago* as an example. Biotechnol Bioeng.

[24] Gonciarz J, Kowalska A, Bizukojc M (2016). Application of microparticle-enhanced cultivation to increase the access of oxygen to *Aspergillus terreus* ATCC 20542 mycelium and intensify lovastatin biosynthesis in batch and continuous fed-batch stirred tank bioreactors. Biochem Eng J.

[25] Krull R, Wucherpfennig T, Esfandabadi ME, Walisko R, Melzer G, Hempel DC, Kampen I, Kwade A, Wittmann C (2013). Characterization and control of fungal morphology for improved production performance in biotechnology. J Biotechnol.

[26] Kowalska A, Boruta T, Bizukojć M (2020). Performance of fungal microparticle-enhanced cultivations in stirred tank bioreactors depends on species and number of process stages. Biochem Eng J.

