# Supplementary material for: Morphological-metabolic analysis in Streptomyces rimosus microparticle-enhanced cultivations (MPEC)
Source: Bioprocess Biosyst Eng. 2024 Apr 25;47(6):891–902. doi: 10.1007/s00449-024-03015-2 (PMC11101530; doi:10.1007/s00449-024-03015-2)
Supplement: Supplementary file 1 — Supplementary file1 (PDF 151 KB) [file 449_2024_3015_MOESM1_ESM.pdf]

Supplementary Material for *Bioprocess and Biosystems Engineering*

Morphological-metabolic analysis in *Streptomyces rimosus* microparticle-enhanced cultivations (MPEC)

Anna Ścigaczewska [a]*, Tomasz Boruta[b], Marcin Bizukojć[c]

*corresponding author: anna.kowalska.1@p.lodz.pl

[a,b,c]Lodz University of Technology, Faculty of Process and Environmental Engineering, Department of Bioprocess Engineering, ul. Wolczanska 213, 93-005 Lodz, Poland

Examined *Streptomyces rimosus* secondary metabolites

Structures of *Streptomyces rimosus* secondary metabolites and their experimental monoisotopic masses at ESI^- ionization.

1. Oxytetracycline (OTC)

Oxytetracycline is a broad-spectrum natural antibiotic from the tetracycline group. This compound is widely used in human and animal treatments, both in the bacterial and fungal diseases [1].

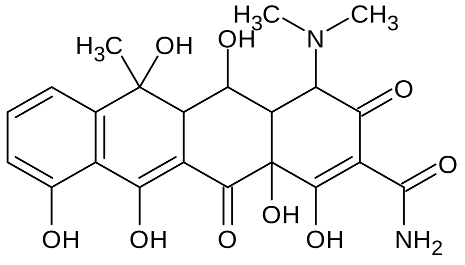

Figure S1 **Oxytetracycline** $C_{22}H_{24}O_9N_2$ $[M\text{-}H]^-$ ion: *m/z*= 459.1427 [Δ(m/z)=+0.0023], t_R=4.36 min.

2. 2-acetyl-2-decarboxamido-oxytetracycline (ADOTC)

2-acetyl-2-decarboxamido-oxytetracycline is an antibiotic, byproduct accompanies a bioproduction of oxytetracycline by *S. rimosus*. It differs from oxytetracycline by the initiation molecule in polyketide synthase [2, 3].

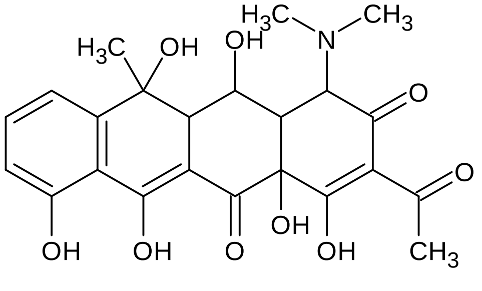

Figure S2 **2-acetyl-2-decarboxamido-oxytetracycline (ADOTC)** $C_{23}H_{24}O_9N_1$ $[M\text{-}H]^-$ ion: m/z=458.1409 [Δ(m/z)=-0.0042], t_R=4.68 min.

3. Rimocidins

Rimocidins are polyene compounds synthesized by actinomycetes. These natural products are valued for their wide range of biological functions, including antifungal activity [4, 5].

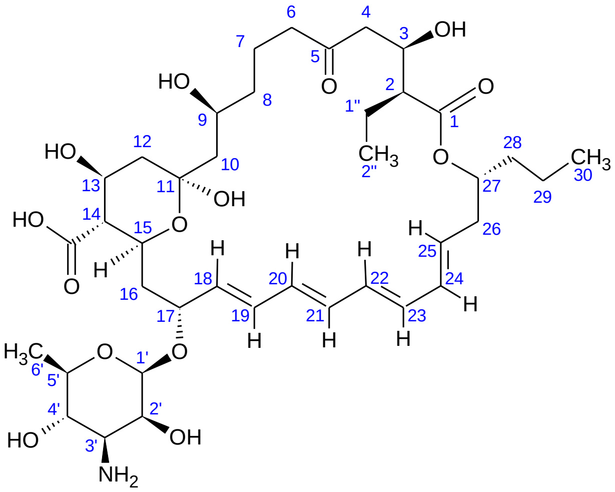

Figure S3 **Rimocidin** $C_{39}H_{61}O_{14}N$ [M-H]$^-$ ion: *m/z*=766.3990 [Δ(m/z)=-0.0024].

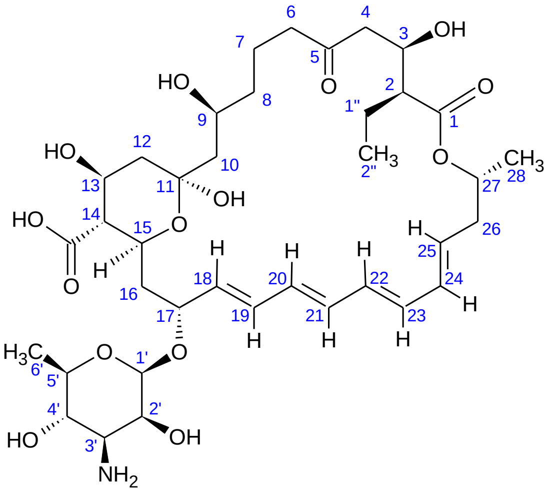

Figure S4 **Rimocidin CE-108** (27-methyl) $C_{37}H_{57}O_{14}N$ [M-H]- ion: m/z=738.3635 [Δ (m/z)=-0.0066].

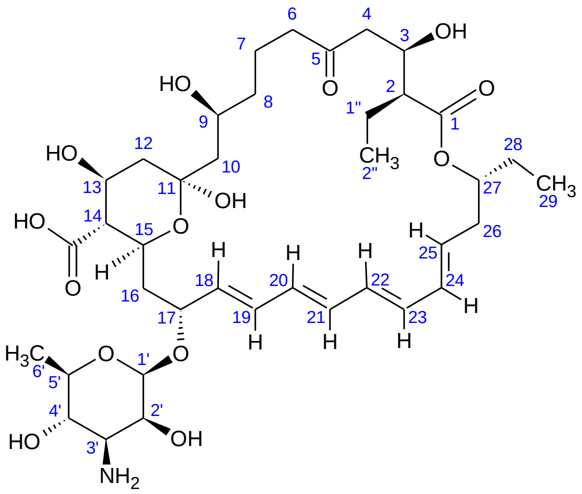

Figure S5 **Rimocidin (27-ethyl)** $C_{38}H_{59}O_{14}N$ $[M\text{-}H]^-$ ion: *m/z*= 752.3879 [Δ(m/z)=+0.0022].

4. Milbemycin derivatives

Experimental m/z value $[M\text{-}H]^-$ ion: *m/z*=591.2823 [Δm/z=+0.000424] for the retention time t_R=6.15 min indicated on the molecule milbemycin A_3 with four additional oxygen atoms and the formula $C_{31}H_{44}O_{11}$. Nakagawa et al. [6, 7] described the possible positions of oxygen atom in the molecule of milbemycin. Taking it into account the found molecule could be either 13β,28, 30-trihydroxy-14,15-epoxymilbemycin A_3 or 13β,26,30-trihydroxy-14,15-epoxymilbemycin A_3 or 13β,26,28-trihydroxy-14,15-epoxymilbemycin A_3 or 26,28,30-trihydroxy-14,15-epoxymilbemycin A_3 or 13β, 26, 28 30-tetrahydroxymilbemycin A_3.

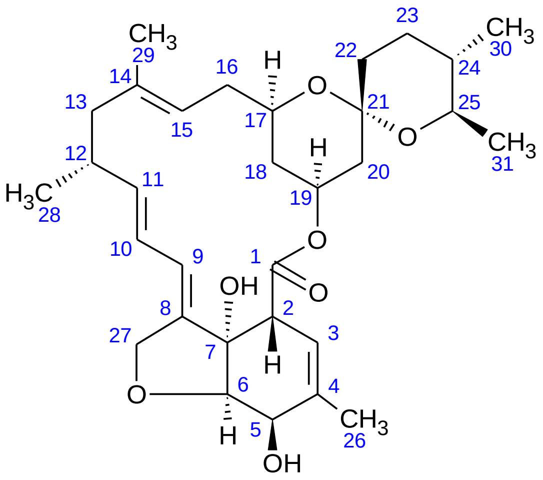

Figure S6 **Milbemycin A$_3$** structure.

Experimental m/z value [M-H]$^-$ ion: *m/z*=593.3038 [Δm/z=+0.00761] for the retention time t_R=6.67 min indicated on the molecule milbemycin β$_{11}$ with four additional oxygen atoms and the formula $C_{31}H_{46}O_{11}$. The most probable compounds described by this formula could be 13β,28,30-trihydroxy-14,15-epoxymilbemycin β$_{11}$ or 13β,26,30-trihydroxy-14,15-epoxymilbemycin β$_{11}$ or 13β,26,28-trihydroxy-14,15-epoxymilbemycin β$_{11}$ or 26,28,30-trihydroxy-14,15-epoxymilbemycin β$_{11}$ or 13β,26,28,30-tetrahydroxymilbemycin β$_{11}$ [8].

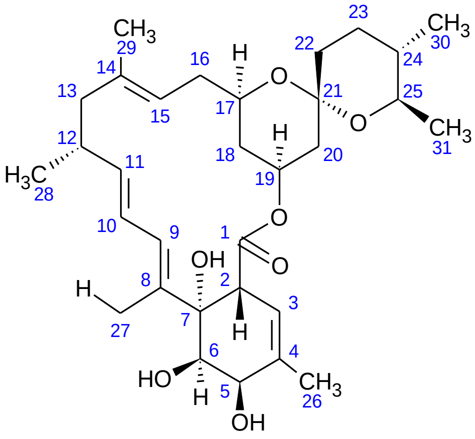

milbemycin b$_{11}$

Figure S7 **Milbemycin β$_{11}$** structure.

5. References

[1] Pickens LB, Tang Y (2010) Oxytetracycline Biosynthesis. JBC. https://doi.org/10.1074/jbc.R110.130419

[2] Hochstein FA, Schach von Wittenau M, Tanner Jr. FW, Murai K (1960) 2-Acetyl-2-decarboxamidoöxytetracycline. J Am Chem Soc. https://doi.org/10.1021/ja01507a034

[3] Lykkeberg AK, Sengeløv G, Cornett C, Tjørnelund J, Hansen SH, Halling-Sørensen B (2004) Isolation, structural elucidation and in vitro activity of 2-acetyl-2-decarboxamido-oxytetracycline against environmental relevant bacteria, including tetracycline-resistant bacteria. J Pharm Biomed Anal. https://doi.org/10.1016/S0731-7085(03)00629-0

[4] Seco EM Perez-Zuniga FJ, Rolon MS, Malpartida F (2004) Starter Unit Choice Determines the Production of Two Tetraene Macrolides, Rimocidin and CE-108 in *Streptomyces distaticus* var 108. Chem Biol. https://doi.org/10.1016/j.chembiol.2004.02.017

[5] Seco EM, Cuesta T, Fotso S, Laatsch H, Malpartida F (2005) Two Polyene Amides Produced by Genetically Modified *Streptomyces diastaticus* var. 108. Chem Biol. https://doi.org/10.1016/j.chembiol.2005.02.015

[6] Nakagawa K, Sato K, Tsukamoto Y, Okazaki T, Torikata A (1994) Microbial conversion of milbemycin: microbial conversion of milbemycins A_4 and A_3 by *Streptomyces libani*. J Antibiot. https://doi.org/10.7164/antibiotics.47.502

[7] Nakagawa K, Sato K, Tsukamoto Y, Torikata A (1994) Microbial conversion of milbemycins: 28-hydroxylation of milbemycins by *Amycolata autotrophica*. J Antibiot. https://doi.org/10.7164/antibiotics.46.518

[8] Nonaka K, Tsukiyama T, Okamoto Y, Sato K, Kumasaka C, Yamamoto T, Maruyama F, Yoshikawa H (2000) New milbemycins from *Streptomyces hygroscopicus subsp. aureolacromosus*: Fermentation isolation and structure elucidation J Antiobiot. https://doi.org/10.7164/antibiotics.53.694
